# Mandibular dental arch short and long-term spontaneous dentoalveolar changes after slow or rapid maxillary expansion: a systematic review

**DOI:** 10.1590/2177-6709.22.3.055-063.oar

**Published:** 2017

**Authors:** Arthur César de Medeiros Alves, Olga Benário Vieira Maranhão, Guilherme Janson, Daniela Gamba Garib

**Affiliations:** 1 Universidade de São Paulo, Department of Orthodontics, Bauru, São Paulo, Brazil.

**Keywords:** Dental arch, Palatal expansion technique, Mandible, Mixed dentition, Permanent dentition.

## Abstract

**Objective:**

The aim of this systematic review was to analyze the short and long-term spontaneous dentoalveolar changes of the mandibular dental arch after slow (SME) or rapid (RME) maxillary expansion in the mixed and early permanent dentitions.

**Methods:**

An electronic search was performed in the following databases: PubMed/Medline, Cochrane Library, Scopus, Embase and Web of Science. Eligibility criteria for article selection included randomized controlled trials and prospective studies written in English, with no restriction of year of publication, involving patients who underwent SME or RME during the mixed or early permanent dentitions. A double-blind search of articles was performed by two reviewers. Initially, the title and the abstract of the studies were read, and their references were also hand-searched for possible missing studies. A methodological quality scoring scale was used to analyze the selected articles.

**Results:**

The search retrieved 373 articles, but only 6 were selected for review after application of the eligibility and exclusion criteria. Non-clinically significant spontaneous dentoalveolar changes of approximately 1mm were found in the mandibular dental arch in the short and long-term, after slow or rapid maxillary expansions. Furthermore, no significant differences were found between treated and control groups.

**Conclusions:**

There is enough evidence to conclude that negligible short and long-term spontaneous dentoalveolar changes tend to occur in the mandibular dental arch after SME or RME in the mixed and early permanent dentitions. More randomized studies with appropriate control group are required to better evaluate this issue.

## RESUMO

**Objetivo:**

o objetivo da presente revisão sistemática foi analisar as alterações dentoalveolares espontâneas, em curto e longo prazos, após a expansão lenta (ELM) ou rápida (ERM) da maxila, durante a dentição mista e permanente jovem.

**Métodos:**

uma busca eletrônica foi executada nas seguintes bases de dados: PubMed/Medline, Cochrane Library, Scopus, Embase e Web of Science. Os critérios de elegibilidade para a seleção dos artigos incluíram ensaios clínicos randomizados controlados e estudos prospectivos escritos em língua inglesa, sem restrição do ano de publicação, envolvendo pacientes que foram submetidos a ELM ou ERM durante a dentição mista ou permanente jovem. Dois revisores realizaram, de forma independente, uma busca por artigos. Inicialmente, o título e o resumo dos artigos foram lidos, e uma busca manual foi realizada nas referências dos artigos selecionados, a fim de se avaliar possíveis estudos não encontrados.

**Resultados:**

trezentos e setenta e três artigos foram encontrados com a busca, porém apenas seis foram selecionados para a revisão após a aplicação dos critérios de inclusão e de exclusão. Alterações dentoalveolares espontâneas sem significância clínica de aproximadamente 1 mm foram encontradas na arcada dentária inferior em curto e longo prazos, após a expansão lenta ou rápida da maxila. Além disso, não foram observadas diferenças significativas entre os grupos tratados e os controles.

**Conclusão:**

existe evidência suficiente para se concluir que alterações dentoalveolares espontâneas sem significância clínica tendem a ocorrer na arcada dentária inferior em curto e longo prazos após a ELM e ERM na dentição mista e permanente jovem. Mais ensaios clínicos randomizados com grupos controles adequados são necessários para melhor avaliar essa questão.

## INTRODUCTION

Maxillary dental arch constriction is commonly associated with unilateral or bilateral posterior crossbites in the mixed or early permanent dentitions.^1^ ^,^ ^2^ Considering that transverse malocclusions do not self-correct during the craniofacial growth, either slow (SME) or rapid (RME) maxillary expansions should be performed as early as possible to transversely increase the maxillary dental arch with a combination of orthopedic and orthodontic effects.^3^ ^,^ ^4^ In general, the greater the patient’s age, the greater the dental effects and the smaller the skeletal changes.^5^

Occasionally, maxillary constriction is not associated to posterior crossbites.^6^ Spontaneous progressive constriction of the mandibular dental arch might occur from childhood to adulthood as an adaptation process to the progressive maxillary constriction observed in untreated patients.^1^ Logically, if maxillary constriction induces mandibular dental arch constriction over time, maxillary expansion might induce spontaneous increase of the mandibular dental arch width in the short or long-term.^7^ This hypothesis is based on the fact that the maxillary dental arch expansion modifies the balance of forces between the tongue and cheek on the mandibular teeth.^8^ Predominance of the tongue forces on the mandibular teeth might increase the mandibular dental arch width.^9^

Spontaneous dentoalveolar changes in the mandibular dental arch concurrent to SME or RME may have clinical implications regarding the indication of mandibular dental arch dentoalveolar expansion. Therefore, randomized^10^ ^-^ ^12^ (RCT) and non-randomized^13^ ^-^ ^15^ clinical trials have been developed to answer this clinical issue. However, no consensus has been reached. Thus, the aim of the present systematic review is to evaluate the short and long-term spontaneous dentoalveolar changes in the mandibular dental arch, after slow or rapid maxillary expansion in the mixed or early permanent dentitions.

## MATERIAL AND METHODS

The protocol of this systematic review was prepared and registered in PROSPERO (CRD42016039760). This review was conducted based on the PRISMA Statement for Systematic Review^16^ and comprised articles available until May of 2016. Eligibility criteria for article selection included randomized clinical trials and prospective studies written in English, with no restriction of year of publication, involving orthodontic patients with 6 to 12 years of age. These patients should present with maxillary constriction in the mixed or early permanent dentitions and should have been treated with slow or rapid maxillary expansion. Evaluation of the spontaneous dentoalveolar changes in the mandibular dental arch should have been performed in the short (3 to approximately 12 months post-expansion) or long-term (more than 12 months post-expansion). At least one of the following variables should have been measured in the mandibular dental arch by means of conventional or digital dental models or posteroanterior radiographs: intercanine distance, inter-deciduous molar or interpremolar distances, inter-first permanent molar distance, arch perimeter, arch length and buccolingual inclination of the canines and posterior teeth. The definition of each variable is shown in Table 1.

**Table 1:** Definition of outcome measurements.

Outcome measurement	Definition
Intercanine distance	Linear distance between the crown tips or the midpoints of the lingual gingival margins of both mandibular canines.
Inter-deciduous molar or interpremolar distances	Linear distance between the buccal cusp tips or the midpoints of the lingual gingival margins of the left and right mandibular deciduous molars or premolars.
Inter-first permanent molar distance	Linear distance between the mesiobuccal cusp tips, the center of the fossa or the midpoints of the lingual gingival margins of both mandibular permanent first molars.
Arch length	A line measured perpendicularly in the horizontal plane connecting the mesial aspects of the mandibular permanent first molars to the point between the mandibular central incisors.
Arch perimeter	The length of a curve from the mesial surface of the mandibular permanent first molars, bisecting the contact points of the deciduous molars or premolars and canines, and smoothly fitting on the incisal edges of the anterior teeth.
Tooth inclination	Angle between the clinical crown axis and the occlusal plane.

The exclusion criteria were patients with oral clefts or associated craniofacial anomalies, previous orthodontic treatment, intervention in the mandibular dental arch during the follow-up period, surgically-assisted rapid maxillary expansion and the lack of a control group.

An electronic search was performed in the following databases with the assistance of a senior librarian specialized in Health Sciences databases: PubMed/Medline, Cochrane Library, Scopus, Embase and Web of Science. The search strategy used in the aforementioned databases included the MeSh terms “dental arch” and “palatal expansion technique” or “maxillary expansion” and “mixed dentition” or “permanent dentition”. 

A double-blind search of articles was performed by two reviewers. Initially, the title and the abstract of the studies found in each database were independently read by both examiners according to PICO. The references of the articles were also hand-searched for possible missing studies. In case of disagreement regarding which article fulfilled the inclusion criteria, consensus was reached by discussion between the two reviewers. The articles that fulfilled the inclusion criteria were included in the systematic review and were qualitatively analyzed using the Cochrane collaboration recommendations^17^ and a modification of the methodological quality scoring scale developed by Vilani et al^18^ (Table 2). This modification was proposed to evaluate both the selected randomized and non-randomized controlled studies. Kappa statistics were used to evaluate the interexaminer agreement after the articles selection and to perform the quality assessment of the final studies.

**Table 2:** Quality assessment scale.

Component	Classification	Points	Definition
Selection bias			
1. Randomization	Adequate	1.0	Randomization correctly described as well the randomization method.
	Inadequate	0.5	Incomplete description of randomization method.
	None	0	No description of randomization method.
2. Allocation concealment	Adequate	1.0	Allocation concealment correctly described.
	Inadequate	0.5	Incomplete description of allocation concealment.
	None	0	No description of allocation concealment.
Performance bias			
3. Blinding of participants and personal	Adequate	1.0	Blinding of participants and personal correctly described and effectiveness of blinding stated.
	Inadequate	0.5	Incomplete description of blinding of participants and personal.
	None	0	No description of blinding of participants and personal.
Detection bias			
4. Blinding assessment	Adequate	1.0	Blinding assessment described in measures or statistics and effectiveness of blinding stated.
	Inadequate	0.5	Incomplete description of blinding assessment.
	None	0	No blinding assessment described.
Attrition bias			
5. Incomplete outcome data	Explained	1.0	Dropouts reported with explanation and description of complete or incomplete data retrieved.
	Not explained	0.5	Dropouts reported with no explanation or description of complete or incomplete data retrieved.
	None	0	No reporting of dropouts or data retrieved.
Reporting bias			
6. Selective reporting	Adequate	1.0	No selective reporting of primary outcomes.
	Inadequate	0.5	Insufficient information to judgement.
	None	0	Selective reporting of primary outcomes.
Other kinds of bias			
7. Eligible criteria for participants	Adequate	1.0	Inclusion/exclusion criteria described.
	Inadequate	0.5	No description of inclusion/exclusion criteria, but selection done at least by age and type of expansion.
	None	0	No description of criteria for selection.
8. Presence of a control group	Yes	1.0	Presence of a control group.
	No	0	Absence of a control group.
9. Statistical treatment	Adequate	1.0	Statistical treatment fully described and adequate.
	Inadequate	0.5	Statistical treatment not fully described or inadequate.
	None	0	No statistical treatment applied.
10. Reliability of measures	Adequate	1.0	Aleatory measures repeated and statistical test applied.
	Inadequate	0.5	Measures repeated and inadequate or no statistical tests applied.
	None	0	Measures not repeated.
11. Potential bias and trial Limitations	Fully	1.0	Description of potential bias and trial limitations acknowledging them.
	Partially	0.5	Description of potential bias and trial limitations without acknowledging them.
	None	0	No description of potential bias or trial limitations.

## RESULTS

**Figure 1:**
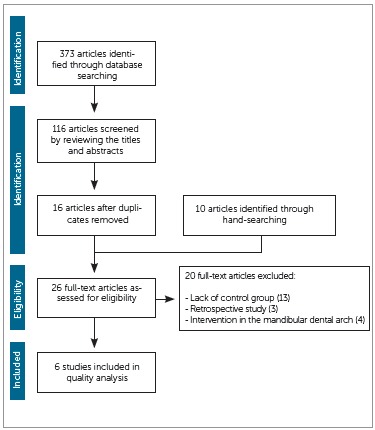
Flow diagram adapted from the PRISMA statement.^16^

The electronic search retrieved 373 articles. After examination of the titles and abstracts of these studies, 56 articles were selected, however this number was reduced to 16 when duplicates were removed. Ten additional articles were found after hand-search on the references of the previous 16 studies found. The full-text copies of all of these articles were analyzed according to the eligibility and exclusion criteria, resulting in 6 studies qualified for the final analysis. The flow diagram shows the process of article selection (Fig 1). Kappa statistic was performed after article selection and showed excellent interexaminer agreement (*K* = 0.94).^19^

A summary of the methodological characteristics of the final studies - such as authors, year of publication, study design, sample size, dentition stage, type of expansion procedure, type of appliance used, amount of maxillary expansion, follow-up period and the measurements performed in the mandibular dental arch - is given in Table 3. Application of the methodological quality check-list is shown in Table 4. Kappa statistics was performed after quality assessment of the studies and showed good interexaminer agreement (0.85).^19^

**Table 3:** Summarized data of the six included studies.

Author	Year	Study design	Sample	Dentition	Expansion procedure	Appliance	Appliances with occlusal coverage	Amount of expansion	Expansion with overcorrection	Follow-up period	Measurements
Bjerklin^13^	2000	Prospective study	60 subjects	Mixed dentition and early permanent dentition	Slow maxillary expansion	Quad-helix and removable expansion plate	No	Not mentioned	Yes	12.5 and 81.9 months for the removable expansion plate; 7.7 and 76.1 months for the quad-helix; 85.8 months for the control group	Mandibular intercanine and inter-first permanent molar distances
O’Grady et al.^14^	2006	Prospective study	66 subjects	Early mixed dentition	Rapid maxillary expansion with and without dentoalveolar expansion of the mandibular dental arch	Acrylic bonded maxillary expander	Yes (Acrylic bonded maxillary expander)	7-8 mm	Yes	38 months	Mandibular intercanine, inter-first premolar, inter-second premolar and inter-first permanent molar distances, arch length and arch perimeter
Cozzani et al.^15^	2007	Prospective study	91 subjects	Mixed dentition	Rapid maxillary expansion	Haas expander	No	6.8 mm	Yes	13 and 28 months	Mandibular intercanine, inter-second deciduous molar and inter-first permanent molar distances
Petrén et al.^10^	2008	Randomized controlled trial	60 subjects	Mixed dentition	Slow maxillary expansion	Quad-helix and removable expansion	No	Not mentioned	Yes	12 months	Mandibular intercanine and inter-first permanent molar distances
Godoy et al.^12^	2011	Randomized controlled trial	99 subjects	Early mixed dentition	Slow maxillary expansion	Quad-helix and removable expansion	No	No	Yes	10.24 and 24.24 months for the quad-helix; 12.12 and 26.12 months for the removable expansion plate; 20 months for the control group	Mandibular intercanine and inter-first permanent molar distances
Petrén et al.^11^	2011	Randomized controlled trial	55 subjects	Mixed dentition	Slow maxillary expansion	Quad-helix and removable expansion	No	Not mentioned	Yes	6 months and 36 months	Mandibular intercanine and inter-first permanent molar distances

**Table 4:** Quality assessment of the selected studies.

	Selection bias		Performance bias	Detection bias	Attrition bias	Reporting bias	Other kinds of bias						
Article	Randomization	Allocation concealment	Blinding of participants	Blinding assessment	Incomplete outcome	Selective reporting	Eligible criteria for participants	Presence of a control group	Statistical treatment	Reliability of measures	Potential bias and trial limitations	Total points	Research quality
Bjerklin^13^, 2000	0	0.5	0	0	0.5	0.5	1	1	0.5	1	0	5	Moderate
O’Grady et al.^14^, 2006	0	0.5	0	0	0	0.5	0.5	1	0.5	1	0	4	Low
Cozzani et al.^15^, 2007	0	0.5	0	0	0.5	0.5	0.5	1	0.5	1	0	4.5	Low
Petrén et al.^10^, 2008	1	1	0	1	1	1	1	1	1	1	1	10	High
Godoy et al.^12^, 2011	1	1	0	1	0.5	1	1	1	1	1	1	9.5	High
Petrén et al.^11^, 2011	0.5	0.5	0	1	1	0.5	1	1	1	1	1	8.5	High

Research quality or methodological soundness: high, >8 points; moderate, 5 to 8 points; low, <5 points.

All the selected studies assessed the spontaneous dentoalveolar changes of the mandibular dental arch after slow or rapid maxillary expansion, performing measurements with digital sliding caliper in conventional dental models. The main variables assessed in these studies were: mandibular intercanine distance^10^ ^-^ ^15^ (between the crown tip or the lingual gingival margin), inter-deciduous molar or interpremolar distance^14^ ^,^ ^15^ (between the center of the fossa or the lingual gingival margin), inter-first permanent molar distance^10^ ^-^ ^15^ (between the mesiobuccal cusp tips, the center of the fossa or the lingual gingival margin), arch length,^14^ arch perimeter^14^ and inclination of the first permanent molars.^14^

In general, the follow-up period of the spontaneous dentoalveolar changes of the mandibular dental arch after SME was greater than that of the RME. The short-term spontaneous dentoalveolar changes after slow maxillary expansion was evaluated in four studies,^10^ ^-^ ^13^ while three of them assessed the long-term effects of SME.^11^ ^-^ ^13^ On the other hand, only one of the selected studies analyzed the short-term spontaneous dentoalveolar changes after rapid maxillary expansion,^15^ while the long-term occlusal changes after RME was analyzed in two studies.^14^ ^,^ ^15^

### Short-term spontaneous dentoalveolar changes after SME

The removable expansion plate promoted greater increases of the mandibular intermolar distance (0.4mm, at the lingual gingival margin level, and 1.2mm, at the mesiobuccal cusp tip level), compared to the quad-helix appliance (-0.4mm, at the lingual gingival margin level, and -0.1mm, at the mesiobuccal cusp tip level), six months after slow maxillary expansion.^11^

No significant spontaneous dentoalveolar changes were observed in the mandibular dental arch of the treated and untreated groups 4.24 months^12^ and 7.7 months^13^ after slow maxillary expansion using the quad-helix appliance and 6.12 months^12^ and 12.5 months^13^ after SME with the removable expansion plate. On the other hand, the quad-helix appliance promoted a significantly greater increase of the mandibular intercanine distance (0.2mm) compared to the control group (0.0mm), 12 months after the expansion procedure.^10^

### Long-term spontaneous dentoalveolar changes after SME

The quad-helix appliance promoted a significantly greater increase of the mandibular inter-first permanent molar distance (0.46mm) compared to the removable expansion plate (-0.36mm), 16.24 to 18.12 months after slow maxillary expansion. However, no significant differences were found between the experimental groups and the control group (-0.18mm).^12^

Thirty-six months after the slow maxillary expansion procedure, the control group showed a significantly greater increase of the mandibular inter-first permanent molar (0.5mm), compared to the quad-helix (-0.6mm) and the removable expansion plate groups (-0.6mm).^11^

Seventy-six months after SME, the quad-helix group showed a significantly smaller decrease (-0.6mm) of the mandibular intercanine distance compared to the control group (-1.3mm).^13^

Finally, a significantly greater increase of the mandibular inter-first permanent molar distance was found for the control group (0.5mm) compared to the removable expansion plate group (-0.1mm), approximately eighty-two months after slow maxillary expansion.^13^

### Short-term spontaneous dentoalveolar changes after RME

Significant increases of 0.9mm in the mandibular intercanine distance and 0.7 mm on the mandibular inter-second deciduous molar distance were found for the experimental group, 1.1 years after rapid maxillary expansion using the Haas-type expander. However, no statistically significant differences were found between the experimental and control groups in the mandibular intercanine and inter-second deciduous molar distances after the 13 months of follow-up. No intra and intergroup differences were found for the mandibular inter first-permanent molar distance.^15^

### Long-term spontaneous dentoalveolar changes after RME

Only a significant increase of 0.9mm was found in the mandibular intercanine distance of the experimental group, 28 months after rapid maxillary expansion with the Haas-type expander.^15^ However, the increase amount in mandibular intercanine, inter-second deciduous molar and in inter-first permanent molar distances were significantly smaller in the experimental compared to the control group.^15^

Similar results were found when spontaneous dentoalveolar changes of the mandibular dental arch were analyzed 3.2 years after rapid maxillary expansion using the acrylic splint expander.^14^ Significant increases of 1.0mm in the mandibular intercanine, 1.8mm in the inter-first deciduous molar, 1.6mm in the inter-second deciduous molar and 1.9mm in the inter-first permanent molar distances were found in the experimental group.^14^ Additionally, significant decreases of 0.8mm in arch length and 1.2mm in arch perimeter and buccal inclination of the mandibular first permanent molars of 7.7° were found in the treated group.^14^ However, no statistically significant differences were found between the experimental and control group changes.^14^

## DISCUSSION

Short and long-term dentoskeletal effects of slow and rapid maxillary expansion were already analyzed in systematic reviews and meta-analyses.^20^ ^-^ ^27^ However, none of these studies evaluated the spontaneous dentoalveolar changes of the mandibular dental arch during these follow-up periods.

A previous study^28^ showed that significant increases of the mandibular dental arch width and perimeter tend to occur 6.1 years after RME in adolescent patients. However, in this longitudinal study, the treatment changes were measured before the expansion procedure and after completion of edgewise appliances therapy. Methodologically, performing orthodontic intervention in the mandibular dental arch represents a confounding factor in the analysis of the spontaneous dentoalveolar changes concurrent to slow or rapid maxillary expansion. Thus, a systematic review of the literature was necessary to better evaluate this issue.

In this review, the selected randomized controlled trials^10^ ^-^ ^12^ obtained greater scores in the quality assessment compared to the prospective studies.^13^ ^-^ ^15^ This finding is expected because the RCTs must be written according to the methodological requirements of the CONSORT statement.^29^ From the randomized controlled trials, the study of Petrén et al^11^ obtained the smallest score because the authors did not describe how the additional ten patients recruited to comprise the treated group, were randomized and allocated.

The selected prospective studies showed important methodological limitations, such as the lack of detailed description of randomization of patients,^11^ ^,^ ^13^ ^-^ ^15^ allocation concealment,^11^ ^,^ ^13^ ^-^ ^15^ blinding assessment,^13^ ^-^ ^15^ reporting of outcome data,^11^ ^,^ ^13^ ^-^ ^15^ drop-out,^14^ ^,^ ^15^ eligibility and exclusion criteria,^14^ ^,^ ^15^ sample size calculation,^13^ ^-^ ^15^ as well as, explanations of the limitations of the study.^13^ ^-^ ^15^ Thus, these studies were qualified with low to moderate risk of bias since the absence of these criteria increases the potential risk of bias.

Neither the selected RCTs^10^ ^-^ ^12^ nor the prospective studies^13^ ^-^ ^15^ reported double-blinding of participants and personal. This is not a methodological concern as double-blinding would not be possible in these experimental studies involving maxillary expansion. Inevitably, both orthodontists and patients were not blinded regarding the appliance type.

The follow-up assessment of treated and untreated patients is important for comparison purposes. However, two studies,^11^ ^,^ ^12^ did not compare the short-term mandibular dental arch changes of the experimental and control groups, because the untreated group was followed-up only in the long-term. Thus, only the experimental group changes could be evaluated in the short-term. The lack of a comparison between the experimental and control group changes in the short-term is a limitation, because it is unknown if the changes observed in the treated group occurred consequently to the maxillary expansion procedure, due to growth, or both.

Additionally, attention is required when interpreting the short and long-term results of Cozzani et al^15^. In this study, the mandibular arch widths in the short and long-term posttreatment observation stages were compared with transversely selected age matching control groups at those stages. Therefore, although there were some spontaneous transverse increases in the experimental group, the mandibular dental arch of the experimental group showed smaller transverse dimensions than the control groups. However, this type of comparison is not very reliable.

The selected studies showed negligible spontaneous dentoalveolar changes of approximately 1mm in the mandibular dental arch of patients treated with slow or rapid maxillary expansion. The clinical implication of this is that clinically significant spontaneous dentoalveolar changes should not be expected in the mandibular dental arch after SME or RME in the short and long-term. If patients show constricted mandibular dental arch with or without incisor crowding, dentoalveolar expansion of the mandibular dental arch may be indicated.^14^ This clinical implication is confirmed by O’Grady et al.^14^ In this longitudinal study, patients treated with the Schwarz’s appliance showed, in the long-term, significantly greater increases of the mandibular dental arch widths, arch perimeter and buccal inclination of posterior teeth compared to the control group.

A limitation of the present systematic review was the difficulty in finding studies with adequate control groups. Additionally, a great number of articles analyze the long-term changes of maxillary expansion followed by fixed orthodontic appliances in the maxillary and mandibular dental arches. Future randomized controlled trials with appropriate control groups should analyze the short and long-term spontaneous dentoalveolar changes of mandibular dental arch after SME and RME to better evaluate this issue. Improvement in the methodological quality of studies and the homogeneity of RCTs might give further information for future meta-analyses, which can provide higher levels of scientific evidence to support the orthodontic clinical practice.^30^

## CONCLUSION

» Based on the results from this systematic review, there is enough evidence to conclude that negligible short and long-term spontaneous dentoalveolar changes occur in the mandibular dental arch after SME or RME in the mixed and early permanent dentition.

» More short and long-term randomized controlled trials with appropriate control groups are needed to better evaluate the spontaneous dentoalveolar changes of mandibular dental arch after slow and rapid maxillary expansion.
